# Mesenchymal Stem Cells (MSCs) Coculture Protects [Ca^2+^]_i_ Orchestrated Oxidant Mediated Damage in Differentiated Neurons In Vitro

**DOI:** 10.3390/cells7120250

**Published:** 2018-12-06

**Authors:** Adel Alhazzani, Prasanna Rajagopalan, Zaher Albarqi, Anantharam Devaraj, Mohamed Hessian Mohamed, Ahmed Al-Hakami, Harish C. Chandramoorthy

**Affiliations:** 1 Department of Internal Medicine, College of Medicine, King Khalid University, Abha 61421, Saudi Arabia; alhazzani@kku.edu.sa; 2 Center for Stem Cell Research, College of Medicine, King Khalid University, Abha 61421, Saudi Arabia; zaheralbarqi2@gmail.com (Z.A.); adevaraj@kku.edu.sa (A.D.); amalhakami@kku.edu.sa (A.A.-H.); 3 Department of Clinical Laboratory Sciences, College of Applied Medical Sciences, King Khalid University, Abha 61421, Saudi Arabia; rajagopalan@kku.edu.sa; 4 Department of Microbiology and Clinical Parasitology, College of Medicine King Khalid University, Abha 61421, Saudi Arabia; 5 Department of Biochemistry, College of Medicine, King Khalid University, Abha 61421, Saudi Arabia; mohamed.hessien@fulbrightmail.org; 6 Department of Chemistry, Division of Biochemistry, Faculty of Science, Tanta University, Tanta City 31512, Egypt

**Keywords:** cerebral ischemia, neuroinflammation, MSCs coculture, MSC rescue, in vitro neuronal differentiation

## Abstract

Cell-therapy modalities using mesenchymal stem (MSCs) in experimental strokes are being investigated due to the role of MSCs in neuroprotection and regeneration. It is necessary to know the sequence of events that occur during stress and how MSCs complement the rescue of neuronal cell death mediated by [Ca^2+^]_i_ and reactive oxygen species (ROS). In the current study, SH-SY5Y-differentiated neuronal cells were subjected to in vitro cerebral ischemia-like stress and were experimentally rescued from cell death using an MSCs/neuronal cell coculture model. Neuronal cell death was characterized by the induction of proinflammatory tumor necrosis factor (TNF)-α, interleukin (IL)-1β and -12, up to 35-fold with corresponding downregulation of anti-inflammatory cytokine transforming growth factor (TGF)-β, IL-6 and -10 by approximately 1 to 7 fold. Increased intracellular calcium [Ca^2+^]_i_ and ROS clearly reaffirmed oxidative stress-mediated apoptosis, while upregulation of nuclear factor NF-κB and cyclo-oxygenase (COX)-2 expressions, along with ~41% accumulation of early and late phase apoptotic cells, confirmed ischemic stress-mediated cell death. Stressed neuronal cells were rescued from death when cocultured with MSCs via increased expression of anti-inflammatory cytokines (TGF-β, 17%; IL-6, 4%; and IL-10, 13%), significantly downregulated NF-κB and proinflammatory COX-2 expression. Further accumulation of early and late apoptotic cells was diminished to 23%, while corresponding cell death decreased from 40% to 17%. Low superoxide dismutase 1 (SOD1) expression at the mRNA level was rescued by MSCs coculture, while no significant changes were observed with catalase (CAT) and glutathione peroxidase (GPx). Interestingly, increased serotonin release into the culture supernatant was proportionate to the elevated [Ca^2+^]_i_ and corresponding ROS, which were later rescued by the MSCs coculture to near normalcy. Taken together, all of these results primarily support MSCs-mediated modulation of stressed neuronal cell survival in vitro.

## 1. Introduction

Neuronal cell damage during cerebral ischemia or stroke is a serious neurological complication that limits survival and/or functional recovery [1]. Neuronal cells are highly vulnerable to many potent oxidants, such as reactive oxygen and nitrogen species (ROS and RNS, respectively). High concentrations of unsaturated fatty acids and low concentrations of oxidant scavengers are other factors that are favorable to catalyze free-radical formations and immature cells contributing to overall stress pathology [2]. The cascade of accumulation or depletion of stress factors is questionable, while, in cerebral ischemia or stroke, one or more of these factors either directly or indirectly predispose neuronal cells to death [3]. Hypoxia/ischemia, drug abuse, chronic use of sedative drugs, ethanol, and/or mechanical trauma are some of the conditions that can aid in irreversible neuronal cell death. Although there are various drugs and therapeutic interventions available for the treatment or management of ischemic damage or stroke, it is noteworthy that survival rate is low even in developed countries [4]. Many detailed studies in the recent past have characterized neuronal cell death well, and therapeutic interventions have had little or no impact on cellular recovery [5]. Cellular protection of damaged neuronal cells by resident populations of neural progenitor stem cells or mobilized progenitors does not offer insight into the failure to repair or protect the cells from death [6,7]. On the other hand, neuronal recovery in neurodegenerative diseases cannot be clearly delineated due to additional factors such as abnormal protein dynamics, fragmentation of neuronal Golgi bodies, impaired axonal transport, and dysfunctional neurotrophins (NTFs) complicating neuron pathology [8]. Oxidative stress is the biochemical mechanism involved in neuronal ischemia and neuronal damage [9]. However, neuronal damage is also a characteristic pathology associated with many noninflammatory-associated neurodegenerative diseases.

The major players in oxidative stress (such as alterations of intracellular calcium homeostasis or ROS burden) need to be better understood in terms of the signals from neuronal cells that would affect resident or mobilized progenitor cells [10]. Ca^2+^ homeostasis, involvement of mitochondrial dysfunction, and impaired bioenergetics are basic physiological mechanisms of neuroinflammation and irreversible neuronal cell death. The classical understanding about mitochondria has been with respect to ROS as the target and source of mitochondrial dysfunction [11]. However, the function of mitochondria as an important intracellular calcium-buffering system aiding in the prevention of neuronal cell death has been very recently demonstrated in many other cellular models [12]. It may be noted that the process of neuroinflammation and apoptotic cell death is non-systemic, spanning simultaneous involvement of cellular entities and multiple factors like genetic, transcriptional, exogenous, and endogenous signals. Our previous experience on the regulation of Ca^2+^ and ROS for cell survival has contributed to the basic understanding of exogenous and endogenous signals affecting neuroinflammation [13]. The increase in the expression of proinflammatory cytokines and their cognate receptors has been well-demonstrated in many neurodegenerative diseases [13] for their role in mediating neuronal loss [14]. Further persistent inflammation in the periphery of non-neuronal cells (such as endothelial cells) has been shown to significantly penetrate the blood–brain barrier, thus further complicating gross pathology [15]. It is well known that, during neuronal ischemia, dopamine is released in a Ca^2+^-dependent process, which, in turn, results in oxidation-associated free-radical generation [16]. ROS (generated due to impaired bioenergetics resulting from proinflammatory cytokines and intermediates from mitochondrial dysfunction, such as α-ketoglutaric acid and/or the precursor of glutamate) act reciprocally to cause the sudden release of Ca^2+^ from Ca^2+^ stores (such as the endoplasmic reticulum). This, in turn, increases [Ca^2+^]_i_ concentration, resulting in high [Ca^2+^]_m_ and loss of mitochondrial membrane potential (ΔΨ_m_), resulting in mitochondrial-induced ROS that add to neuronal cell damage.

Though all these stress factors are initiated by proinflammatory cytokines, working in parallel to induce neuronal cell death, it may be noted that many cytokines have been associated with neuroprotection by modulating neuronal cell death. Further, we cannot neglect to mention the role of cytokines, along with pro-survival progenitor stem cell-mediated rescue, which has been the recent subject of extensive investigation in various disease models. There are very few studies particularly addressing mesenchymal progenitor (MSC) rescue of cortical neurons by protein kinase B (Akt) dependent antiapoptotic pathways [17]. Another interesting observation is that damaged mitochondria initiate MSCs to function in an antiapoptotic manner, which further formed the basis of our inclusion of Ca^2+^ and ROS to examine MSCs rescue functions [18]. The importance of intracellular calcium [Ca^2+^]_i_, ROS, and proinflammatory cytokines in the context of ischemic neuronal cell death and their rescue by MSCs need to be understood, and whether there is a coordinated direct relationship existing for cellular pathology and its reversal needs to be investigated. Therefore, in the current study, we investigated neuronal cell death mediated by elevated [Ca^2+^]_i_ levels and oxidative stressors such as ROS and proinflammatory cytokines that are modulated by MSCs cocultures in vitro.

## 2. Materials and Methods

The study was undertaken at the Center for Stem Cell Research and Department of Microbiology and Clinical Parasitology, College of Medicine, King Khalid University, Abha, Saudi Arabia. Ethical clearance was obtained from the King Khalid University ethical committee, College of Medicine, approval letter REC #2015-03-07 for the collection of umbilical-cord blood. SH-SY5Y (ATCC CRL-2266) cells were grown and sub-cultured with DMEM/F12 media supplemented with 10% fetal bovine serum, 2 mM of glutamine, and 1× penicillin and streptomycin. Cells were maintained at 37 °C at 5% CO_2_ and 95% humidity in a CO_2_ incubator.

### 2.1. Design of the Study

The neuronal differentiated SH-SY5Y cells were subjected to stress by oxygen glucose deprivation method. The hallmark factors that skew the neuronal cells to the stress mediated apoptotic death along with functional serotonin release were evaluated. MSC co-culture was attempted to rescue the skewed neuronal cells from the apoptotic death and possible functional restoration.

### 2.2. Neuronal Differentiation of SH-SY5Y Neuroblastoma Cells

We used the modified retinoic acid (RA) method described elsewhere [19] to differentiate SH-SY5Y cells into adult neurons. Briefly, 1 × 10^5^ low passage [7,8,9] SH-SY5Y cells were plated in a 35 mm^2^ tissue culture-treated dish in 2 mL basal growth media and incubated overnight for adherence. On day 1, the cells were washed once with 1 × PBS and replenished with 2 mL of differentiation media 1, which contained basal media with 2.5% FBS and 10 µM RA. Media were changed every other day. The cells were split on day 7 and re-plated with 2 mL of differentiation media 1. On day 8, cells were replenished with 2 mL of differentiation media containing basal media supplemented with 1% FBS and 10 µM RA. Cells were split and re-plated on polylysine-coated dishes on day 10, while 2 mL of differentiation media 3, which was made up of neurobasal media supplemented with 50 ng/mL recombinant nerve growth factor (rNGF), 50 ng/mL brain-derived neurotropic factor (BDNF), 1× B-27 supplement, 20 mM KCl, 2 mM glutamine, 1× pen/strep, 2 mM dibutyryl-cyclic AMP (db-cAMP), and 10 µM RA, was added on day 11. The media wash was changed every other day until day 17 or 18. Differentiated cells were maintained in differentiation media 3 until the experiments. Differentiated neurons were confirmed by staining with anti-Tuj1 antibody 1:100 dilution (MA1-118, Invitrogen, Thermo Fisher, Waltham, MA, USA) followed by secondary staining with Alexa Fluor 488 conjugated anti-mouse IgG (A32723, Invitrogen, Thermo Fisher, Waltham, MA, USA) and anti-NeuN antibody (ab190565, Abcam, Cambridge, UK) at a dilution of 1:2000. The stained images were acquired by Nikon Epi-Fluorescence Microscope (Nikon, Tokyo, Japan) and processed by NIS-Element imaging software (v4.0, Nikon, Tokyo, Japan).

### 2.3. Scratch Assay 

The functional differentiation of neuronal cells was confirmed on day 18 with a simple scratch assay to check the migration pattern. Nonproliferating and diminished migration are the differentiation hallmark in adult neurons. Briefly, a scratch was made on day 18 on confluent cells undergoing differentiation with a 100 µL sterile tip and incubated for 24 h. The migrated cells were enumerated and compared with non-migrated cells. Inhibition migration marks neuronal differentiation [20].

### 2.4. Human Umbilical-Cord Blood Mesenchymal Stem Cells (UCBMSCs)

Our lab has long been using MSCs from umbilical-cord blood and we have a standardized protocol for MSC isolation. Briefly, mononuclear cells from 11 pooled UCB samples were isolated by using Ficoll-Paque (GE Healthcare Life Sciences, Milan, Italy) density gradient centrifugation. After overnight incubation, adherent cells were further washed with 1× PBS and cultured for 2 weeks in a MesenPRO RS medium (Gibco, Thermo Fisher, Waltham, MA, USA) at 37 °C, in 5% CO_2_ in a humidified atmosphere. Post confluence, UCB MSCs were enriched for CD73, CD29, and CD105 using positive selection magnetic-cell sorting, and purity was enumerated by acquiring in Beckman Coulter Novus flow cytometer (Indianapolis, IN, USA). The purity is determined by presence MSCs which are positive for CD105 and CD90 expression, and negative for CD34 and CD45 [21].

### 2.5. In Vitro Cerebral Ischemia

The in vitro cerebral ischemia microenvironment characterized by hypoxia and hypoglycemia was induced by the oxygen-glucose deprivation method carried out for a specific period of time, described elsewhere [22]. Briefly, on day 18, neuronal differentiated SH-SY5Y cells were grown overnight in 24-well tissue-culture plates. The cells were briefly washed with HBSS buffer and replenished with glucose-free basal media pre-bubbled with 100% N_2_ for 30 min. The plates were sealed with Vaseline to prevent exchange of gases and incubated at 37 °C for 5 h. Reperfusion, on the other hand, is the restoration of the regular culture conditions of 37 °C at 5% CO_2_ and 95% humidity, with normal neuro-growth media supplemented with growth factors for 24 h.

### 2.6. In Vitro Differentiated Neuronal Cells and MSCs Coculture Experiments 

Briefly, the differentiated neuronal cells grown in 24-well plates were subjected to stress by the oxygen-glucose deprivation method as described above. Post-ischemic stress, the cells were rescued by plating 1.5 × 10^4^ MSCSs on the cell-culture insert (Nunc 24-well plate insert, 0.4 µM pore size) and incubating for 24 to 48 h. During reperfusion, DMEM conditioning media containing 10% FBS and 1× pen/strep were used.

### 2.7. Enzyme Linked Immunosorbent Assay (ELISA)

Proinflammatory (TNF-α, L-1β and IL-12) and anti-inflammatory (TGF-β, IL-6, and IL-10) cytokines (R and D systems, Minneapolis, MN, USA and Elab science, Houston, TX, USA), and serotonin (ab133053, Abcam, Cambridge, UK) were assessed by ELISA as per manufacturer instructions. Briefly, cell-culture supernatants from the control, stressed, and MSCs coculture experiments were subjected to the ELISA. Background cytokine and serotonin levels were separately assessed only from MSCs that were subtracted for coculture experiments.

### 2.8. Cell-Death Assay 

The cell-death assay was carried out by staining 10^5^ cells each from the experimental and control groups with 7-amino-actinomycin D (7-AAD), CD 73 PE (for the MSCs coculture group) as per standard surface-staining protocol. The cells were then immediately acquired using a BC Novus flow cytometer (Indianapolis, IN, USA) and data were analyzed using Beckman Coulter Kaluza software (v1.2, Brea, CA, USA). The CD73+ve cells were negatively excluded, and then neuronal cell death was evaluated. Quantification was expressed as percent (%) using Graph Pad Prism V (v5.0, San Diego, CA, USA). 

### 2.9. Real-Time PCR

Antioxidant enzymes superoxide dismutase 1 (SOD1), catalase (CAT), and glutathione peroxidase (GPx) were assessed at mRNA levels using real-time PCR. Real-Time Taq Man Gene Expression Assays from Applied Biosystems (Foster City, CA, USA), SOD1 (assay ID Hs00166575_m1), CAT (assay ID Hs00937395_m1), GPx (assay ID Hs00829989_gH), human β-actin (assay ID: Hs99999903_m1), for neuronal differentiation, neuronal nuclei NeuN (assay ID Hs01370653_m1 and Glyceraldehyde 3-phosphate dehydrogenase GAPDH (assay ID Hs03929097_g1) were used as per standard instructions. Briefly, RNA was isolated from the control, stressed, and MSCs-cocultured cells using the RNeasy Mini Kit (Qiagen, Venlo, The Netherlands). Two-hundred nanogram RNA was used for subsequent cDNA synthesis with the High-Capacity cDNA Reverse-Transcription Kit (Applied Biosystems, Foster City, CA, USA). PCR amplification was carried out in triplicates using BioRad Real-Time detection system (Bio-Rad Laboratories, Hercules, CA, USA). mRNA expression levels were normalized to endogenous control human β-actin for antioxidant enzymes and GAPDH for NeuN. Reactions with no cDNA templates served as negative controls. Relative expression was calculated with the double-delta Ct method and data plotted with Graph Pad Prism V (v5.0, GraphPad Software, San Diego, CA, USA). All experiments included negative controls containing no cDNA template.

### 2.10. Annexin V Assay 

The early and late apoptotic cell accumulation assay was performed using an Annexin V Detection Kit from e-Biosciences (Thermo Fisher Scientific, Waltham, MA, USA) as per manufacturer instructions. Briefly, the detached cells from the experimental and control groups were incubated with 0.25 µg/mL Annexin V reagent in 1x binding buffer for 15 min followed by 2 washes with a wash buffer. Cells were resuspended in the binding buffer containing 0.5 µg/mL propidium iodide, 10,000 events were acquired immediately using Beckman Coulter Novus Flow Cytometer (Indianapolis, IN, USA) and data were analyzed using Kaluza software (v1.2, Brea, CA, USA). Early- and late-phase apoptotic cells were segregated with a quadriplot graph and the total percentage of apoptotic cells was represented using Graph Pad Prism software V (v5.0, GraphPad Software, San Diego, CA, USA).

### 2.11. [Ca^2+^]i_c_ Assay

A Fluo-4 NW Calcium Assay Kit from Molecular Probes Cat #F36206 Thermo Fisher Scientific (Waltham, MA, USA) was used to assess the intracellular calcium. Briefly, the differentiated neuronal cells at all experimental conditions (control, stress, and MSCs coculture) were removed from the 24-well experimental format by gently using a rubber policeman. Then, the cells were transferred at a concentration of 10^5^ cells on 96-well tissue culture plates and left for 4 h to adhere. After incubation, the medium was removed and the cells were incubated for 45 min at 37 °C in 100 μL of a loading dye. The plates were read with the spectrometer on a setting of 494 nm excitation and 516 nm emission. The experiments were performed in tetrads. The data were analyzed and plotted using Graph Pad Prism 5 software (v5.0, GraphPad Software, San Diego, CA, USA) [23].

### 2.12. ROS Assay

ROS were evaluated by flow cytometry. Briefly, 10^5^ from the control, stressed, and MSCs coculture cells were each stained with 10 μM 2′,7′-dichlorofluorescein diacetate (H2DCF-DA) (Molecular Probes Cat #D399 Thermo Fisher Scientific, Waltham, MA, USA), as per manufacturer instructions. The cells were acquired immediately using a Beckman Coulter Novus flow cytometer (Indianapolis, IN, USA), and data were analyzed using Beckman Coulter Kaluza software (v1.2, Brea, CA, USA). Quantification was expressed as a percentage using Graph Pad Prism V (v5.0, GraphPad Software, San Diego, CA, USA) [24].

### 2.13. Western Blot 

The neuronal differentiated control, stressed, and MSCs coculture cells were lysed using a radioimmunoprecipitation assay buffer (RIPA) buffer (50 mM Tris–HCl (pH 7.4), 150 mM NaCl, 0.25% deoxycholic acid, 1 mM EDTA, 1% NP-40, protease inhibitor cocktail (complete; Roch and 1 mM PMSF)). Ten micrograms of total protein was subjected to SDS–PAGE and transblotted onto a nitrocellulose membrane, blocked with 3% bovine albumin serum in Tris buffered saline (pH 7.4) with 0.1% Tween 20 (TBS-T) for 2 h and incubated overnight at 4 °C with antibody against anti-NFκB, COX–2, and β-actin (Sigma–Aldrich, St. Louis, MO, USA). Band quantification was performed using Image J (1.8.0, NIH, WI, USA) and normalized with β-actin. 

### 2.14. Statistical Analysis

Unless specified, laboratory experimental data were obtained from a minimum of 3 repeats at different time periods. The data are expressed as mean ± SE, and statistical significance was evaluated via one-way analysis of variance (ANOVA), Student’s *t*-test, and nonparametric Kruskal–Wallis test. A *p* value of <0.05 was considered statistically significant. Data were plotted with Graph Pad Prism (v5.0, GraphPad Software, San Diego, CA, USA).

## 3. Results

**Figure 1:**
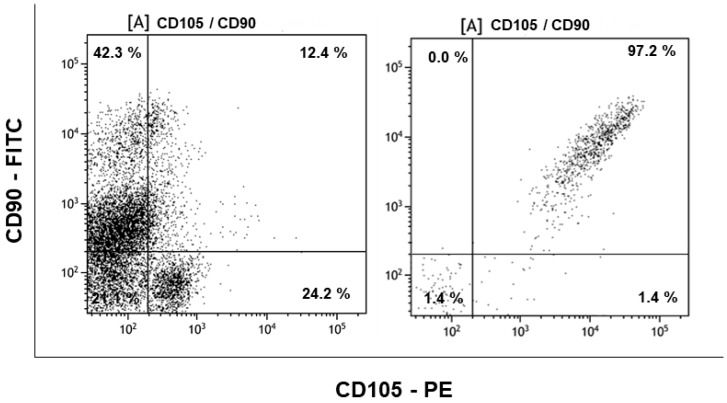
Purity and enrichment of umbilical-cord mesenchymal stem cells (MSCs) using positive selection for CD90/CD105/CD73 cocktail and subjected to phenotypic identification using anti CD105 antibody tagged with Phycoerythrin (PE) in the x-axis and anti CD90 antibodies labelled with Fluorescein isothiocyanate (FITC) flurochormes in y-axis. The left figure is pre-positive selection showing only 12.4% of CD90/CD105 double positive cells in the mononuclear fraction isolated from umbilical-cord blood and the right figure shows the enrichment of about 97.2% CD90/CD105 double positive MSCs after positive selection.

**Figure 2:**
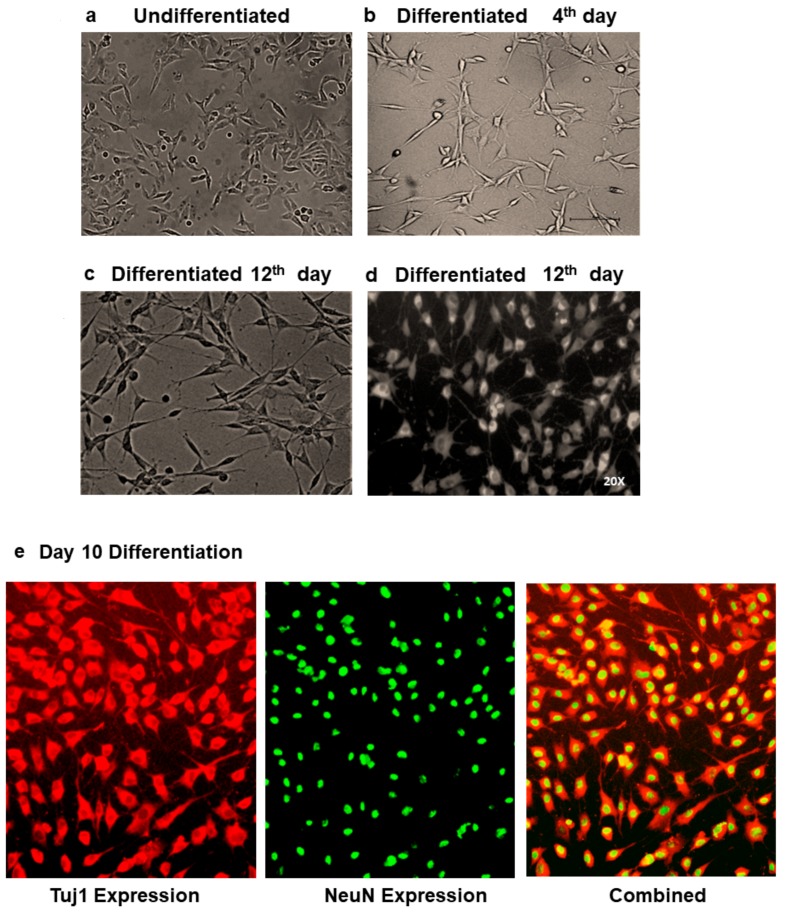

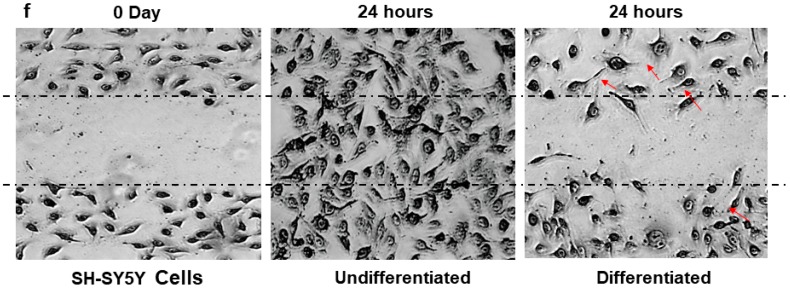
Characterization of neuronal differentiation. (**a**) Undifferentiated SH-SY5Y cells. (**b**–**d**) Differentiation of SH-SY5Y cells showing 4th day and 12-day differentiation. Day 12 differentiation is shown in two modes for better appreciation of the neurite growth. (**e**) Expression of neuronal differentiation marker Tuj1 and NeuN in differentiated cells. The first picture shows the expression of Tuj1 followed by NeuN nuclear expression and combined Tuj1 and NeuN. (**f**) Scratch assay showing stunted migration of differentiated SH-SY5Y cells to adult neuronal cells. Migration was assessed from day 18 differentiated re-plated cells.

In the current study, the MSCs pooled from umbilical-cord blood (n = 11) were isolated by CD105 + CD73 + CD29 positive selection, which gave a purity of more than 97% (Figure 1). Absence of the CD34 and CD45 positive cells in the sorted fraction proved that the cells used were a homogeneous suspension of MSCs (Supplementary Figure S1). These cells were used for the coculture experiments. Functional differentiation of neuronal cells from SH-SY5Y cells was demonstrated with morphological changes (Figure 2A–D). The differentiated cells were confirmed with expression of Tuj1 and NeuN (Figure 2E and Supplementary Figure S2B) and mRNA expression of NeuN in the differentiated cells (Supplementary Figure S2B). The neuronal-differentiated cells showed diminished or no migration, when compared to the neuroblastoma phenotype of the original SH-SY5Y cells. Cessation of migration determined by a simple scratch assay (Figure 2F) further confirmed the neuronal differentiation.

### 3.1. MSCs Coculture Alleviates Neuronal Ischemia Characterized by NF-κB-Mediated Proinflammatory Cytokines

**Figure 3:**
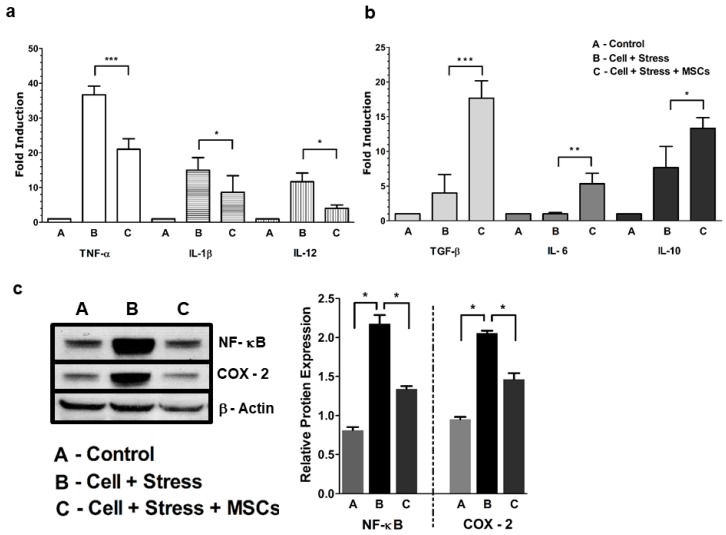
Inflammatory mediator status pre- and post-MSCs coculture. (**a**) Levels of elevated TNF-α, IL-1β, and IL-12 during stress and rescued post-MSCs coculture. (**b**) Anti-inflammatory TFG-β, IL-6, and IL-10 cytokine status during stress and post-MSCs coculture. (**c**) Left panel shows Western blot of NF-κB, COX-2, and β-actin expression. Right panel is blot-band quantification using Image J densitometry. Relative protein expression of NF-κB and COX-2 of the stressed group are compared with the control and rescue groups. Bar represents mean ± SEM, * *p* < 0.05, ** *p* < 0.01, *** *p* < 0.001; significant; *n* = 3.

We first investigated whether proinflammatory cytokines were elevated in neuronal cells under stress. Although many studies have significantly demonstrated the role of cytokine status in inflammation, we felt that, depending upon the mode of stress acquisition, the role and, thereby, the levels of pro and anti-inflammatory cytokines may differ. Based on the results, it was evident that many of the assessed proinflammatory and anti-inflammatory cytokines (Figure 3A,B) in the post stress culture supernatant were either elevated (~35-fold for TNF-α 15-fold for IL-1β and 11-fold for IL-12) or diminished (TGF-β 4-fold, IL-6 1-fold, and IL-10 ~7-fold) respectively compared to controls. The rescue of characteristic inflammatory phenotype was restored with MSCs coculture This resulted in significantly upregulated (Figure 3B) anti-inflammatory cytokines (17-fold for TGF-β 4-fold for IL-6, and 13-fold for IL-10,) and considerably downregulated proinflammatory cytokines (35- to 21-fold in TNF-α from 15- to 8-fold in IL-1β and from 11- to 4-fold in IL-12 (Figure 3A) as observed.

We then checked whether higher levels of proinflammatory cytokines were mediated by NF-κB activation. Our Western-blot results showed elevated NF-κB (Figure 3C) expression when compared with the control cells. In order to substantiate inflammation associated with the NF-κB pathway, we measured COX-2 expression, an important proinflammatory enzyme promoting neuroinflammation and pain. We observed one-fold increase in the expression of COX-2 when compared to the control (Figure 3C). Decreased expression of NF-κb and COX-2 was observed in stressed cells, when cocultured with MSCs indicating the rescue of anti-inflammatory cytokines. Results showed that higher cytokine expression coincided well with elevated NF-κB and COX-2 expression during cellular stress and their rescue with MSCs coculture.

### 3.2. Inflammatory Neuronal Cell Death Characterized by Low Antioxidant Enzymes and Free-Radical Increase are Rescued by MSCs Coculture 

**Figure 4:**
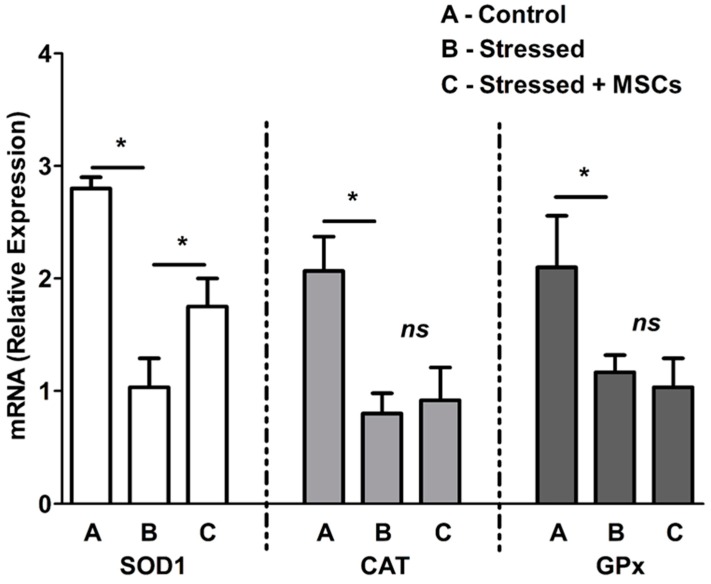
Antioxidant enzyme expression in control (**A**), stressed neuronal cells (**B**) and stressed neuronal cells + MSCs coculture rescue (**C**). The mRNA expression of SOD1, CAT, and GPx was normalized with internal housekeeping gene β-actin and compared to the control. Values are expressed as mean ± SEM, * *p* < 0.05, significant; *n* = 3.

**Figure 5:**
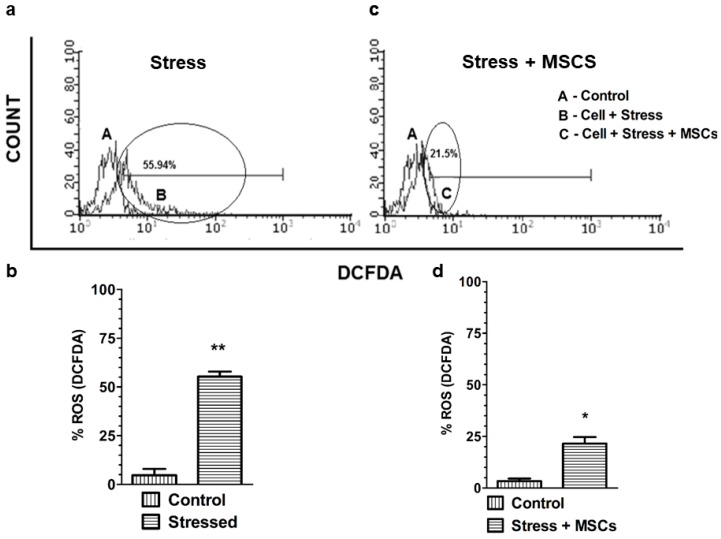
Elevated reactive oxygen species (ROS) characterize proinflammation. (**a**) ROS status of control (A) and stressed neuronal cells (B). (**b**) ROS quantification in the control (A) and stressed neuronal cells (B). (**c**) ROS levels in the control (A) and stressed neuronal cells + MSCs coculture rescue (C). (**d**) ROS quantification in the control (A) and stressed cells + MSCs coculture rescue (C). Bars represent mean ± SEM, * *p* < 0.05, ** *p* < 0.01; significant; *n* = 3–5.

We next investigated the impact of proinflammatory cytokines on antioxidant enzymes and ROS. mRNA expression of antioxidant enzymes SOD1, GPx, and CAT at cellular levels (Figure 4) were determined. Though the lower expression of antioxidant enzymes under stress were observed and well correlated with increased proinflammatory cytokines and factors (NF-κB and COX-2), the MSCs coculture experiments showed significant higher SOD1 expression with no effect on CAT and GPx. Since only SOD1 responded to MSCs coculture rescue, we next measured the cellular ROS. Our results showed 55.94% ROS accumulation in cells under stress (Figure 5A,B), while a reversal or significant reduction of ROS to 21.5% (Figure 5C,D) was observed with rescue experiments.

**Figure 6:**
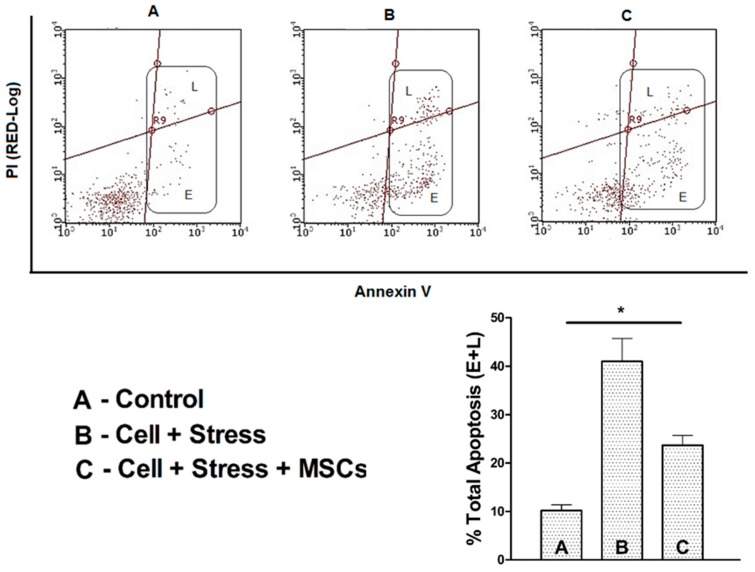
Annexin V/PI assessment of early and late apoptotic cells shown as flow quadrants. Lower figure shows the percentage of early- and late-phase cells in the experimental groups of (**A**) control (**B**) stressed neuronal cells and (**C**) stressed neuronal cells + MSCs coculture rescue. Values are expressed as mean ± SEM, * *p* < 0.05 significant; *n* = 3.

**Figure 7:**
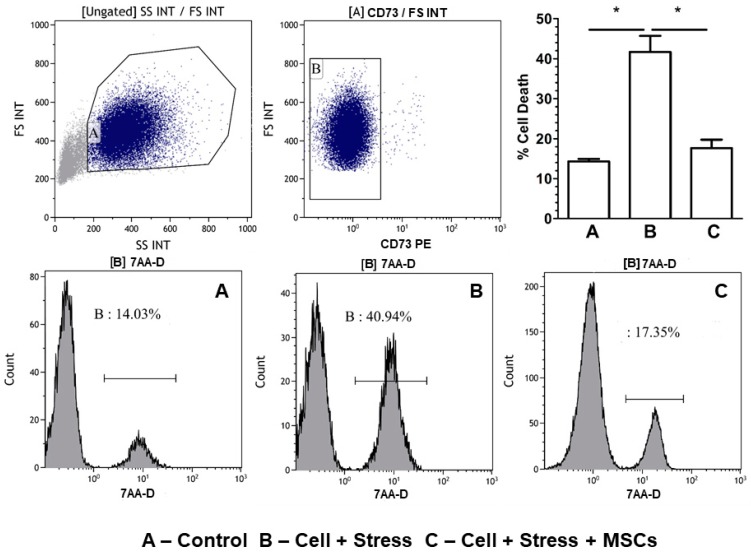
Cell-death assessment using 7-aminoactinomycin D [7-AAD]. The first two flow histograms, from left to right, are the gating strategy used for segregating the MSCs that would have squeezed through the insert into the coculture experiments. MSCs are excluded from total cell death by negative gating and exclusion. Lower histograms show the cell death percentage of the experimental groups (**A**) control (neuronal cells without stress), (**B**) stressed (neuronal cells which has been under stress), and (**C**) MSC coculture (the rescue of the stressed neuronal cells by coculturing MSC through insert). Quantification and percent cell death are shown in the upper-right corner figure. Bars represent mean ± SEM, * *p* < 0.05, significant; *n* = 3.

Further, to substantiate ROS accumulation is detrimental leading to apoptotic neuronal cell death, we measured apoptosis and cell death in two different experiments to clearly delineate the cells that undergo apoptosis and eventually die. The results of annexin V/PI staining showed around 45% accumulation of early (E)- and late (L)-phase apoptotic cells in the stressed group (Figure 6) which was reduced to ˂25% (E + L) apoptotic cells upon MSCs coculture. Subsequently, the percent cell death in the stress group, which was 40.94%, diminished to 17.35% in the rescue indicating cell death through apoptosis (Figure 7A–C).

### 3.3. Elevated Serotonin Release as Altered Neuronal-Cell function is Resuced Upon [Ca^2+^]_i_ Regulation by MSCs Coculture

**Figure 8:**
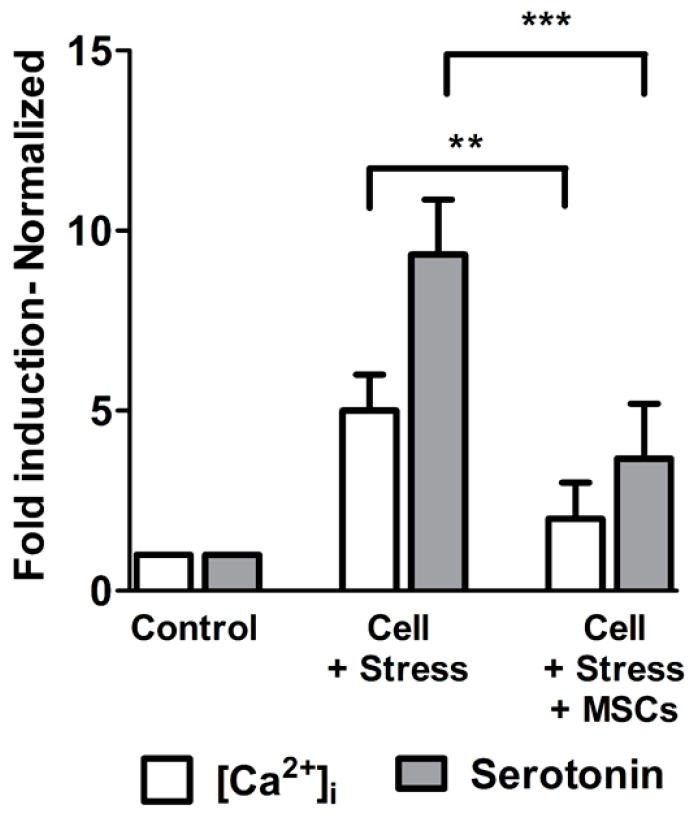
Intracellular calcium concentration and serotonin release were parallel. Increase in [Ca^2+^]_i_ during stress resulted in high serotonin release which was significantly reduced with MSCs rescue. Depiction shows that [Ca^2+^]_i_ regulates serotonin release with experimental groups in x-axis against the quantitative levels of Ca^2+^ and Serotonin expressed as folds in y-axis. Values are expressed as mean ± SEM, ** *p* < 0.01, *** *p* < 0.001; significant; *n* = 3.

The increase in cellular ROS by 55.94% in stressed cells (Figure 5A,B) was well-characterized by five-fold-increased basal [Ca^2+^]_i_ (Figure 8) and about nine-fold corresponding serotonin exocytosis. Altered neuronal function during neuroinflammation is characterized by ROS mediated [Ca^2+^]_i_ dependent serotonin release. The ROS source that was detrimental and promoted apoptosis was dependent on abnormal Ca^2+^ homeostasis observed during cellular stress. Indeed, neuronal cell functions during ischemic stages need to be evaluated in order to understand neuronal-cell function limitations. Therefore, we measured [Ca^2+^]_i_ and serotonin levels, which become calcium channel-dependent processes since an increase in [Ca^2+^]_i_ skews the cells to release serotonin by exocytosis. Our results clearly showed five-fold elevation in [Ca^2+^]_i_ levels during stress (Figure 8), while the same was reduced significantly in MSCs cocultures, to three-fold. Similarly, high serotonin levels correlated with [Ca^2+^]_i_ while gradual restoration of [Ca^2+^]_i_ levels during the MSCs coculture caused a significant reduction in serotonin exocytosis down to four-fold in culture supernatants (Figure 8).

## 4. Discussion

It was demonstrated by some important studies that neuronal cell death during cerebral ischemia occurs via cytokine-mediated transcriptional regulation of inflammatory signals. Upregulation of inflammatory cytokines results in an increase in [Ca^2+^]_i_ and [Ca^2+^]_m_, which facilitate ROS of both general and mitochondrial origin to skew the cell toward alterations of cellular bioenergetics, including apoptosis [25]. Although the central dogma of neuroinflammation is well understood, functional restoration of neuronal cells has always been questioned. In the current study, we intended to restore neuronal cell function by coculturing them with MSCs, which are known for a variety of modulatory functions. The standard retinoic acid protocol for the differentiation of SH-SY5Y cells to an adult neuronal cell type worked well, and results were consistent with already published reports [26,27,28,29]. Instead of assessing neuronal differentiation markers, we functionally determined the transformation into adult neuronal cells by checking their ability to migrate since neuroblastoma is well known to migrate. At the end of retinoic acid differentiation, cells that had morphologically changed to neuronal structures did not migrate, which was in agreement with other published studies.

The very purpose of the current investigation was to therapeutically manage neuroinflammation that occurs as part of cerebral ischemia or stroke. As MSCs are known for modulating pathological tissue to rescue phenotypes [30], we used MSCs cocultures in restoration of neuronal stress in ischemic conditions.

### 4.1. Glucose-Oxygen Deprivation Model Induced NF-kB-Mediated Inflammation in Neuronal Cells

NF-κB is ubiquitously expressed in neurons, and activation is associated with the processing of neuronal information [31]. We know that NF-κB is activated by varied responses, including inflammation [32]. It is clear from our results that inflammation is characterized by elevated proinflammatory cytokines, such as TNF-α and IL-1β and -12. At the molecular level, NF-κB-mediated neuroinflammation is known to transcriptionally regulate the expression of proinflammatory cytokines and induce expression of proinflammatory enzymes such as cyclooxygenases (COX-2). The higher expression of NF-κB, proinflammatory cytokines, and enzymes in the stressed group was in agreement with reports already published in the literature [33,34,35]. The purpose of adding COX-2 with NF-κB was to demonstrate that the latter regulates the expression of the former by adding more proinflammatory factors at membrane levels [34]. From our results, it was evident that COX-2 expression had a proportional increase with NF-κB, as reported by other studies, which implicates its role in neuroinflammation in the brain rather than in the periphery. Many studies have reported upregulated expression of COX-2 during neuronal stress, which was in agreement with our observations that NF-κB largely regulates proinflammatory enzymes, along with cytokines and other cellular factors [36,37,38,39].

Inhibition of anti-inflammatory cytokines (TGF-β and IL-6 and -10) in the stressed groups further confirmed that inflammation induced by glucose-oxygen deprivation is mediated through NF-κB pathways. In addition, induction of early and late apoptosis and enhanced cell death in stressed cells need to be examined, as cell-death turnout was less compared to the 70% accumulation of both the early- and late-phase apoptotic cells. We further speculate that necrotic death can also occur as an alternative pathway with the same factors inducing apoptosis (necroptosis), while the induction of autophagy as a survival mechanism [40] could also be the reason for the accumulation of later-phase apoptotic cells as observed in our study. Furthermore, it should be noted that neurons could undergo diverse forms of cell death in response to varied stress signals.

### 4.2. High ROS Levels Characterized by Elevated [Ca^2+^]_i_ Skew Neuronal Cells to Apoptosis

ROS accumulation is the major player in skewing cells toward death. Normally, physiological ROS levels are continuously produced in neuronal cells and are balanced by endogenous free radical-scavenging enzymes. However, excessive ROS (both of global and mitochondrial origin) during stress can be detrimental to neuronal cells [41]. Excessive ROS are further attributed to increased [Ca^2+^]_i_ levels released by the calcium store (endoplasmic reticulum (ER)) during inflammation [42]. Our previous publications on cellular and bioenergetics models [21,43,44] have shown the increase in basal [Ca^2+^]_i_ that, in turn, increases ROS production, as observed in our results. This may be due to mitochondrial Ca^2+^ buffering action, which, in turn, resulted in ΔΨ_m_ depolarization, promoting ROS production and subsequent apoptosis [43]. Accumulation of early and late apoptotic cells observed in our results was well in agreement with this logic, in which ROS mediate apoptosis. Nonetheless, it is known that Ca^2+^ is a second messenger that mediates intra- and extracellular signals, which physiologically control cellular activity [45]. The interaction of Ca^2+^ with other signaling cascades, such as ROS, at subtoxic levels constitutes several normal cellular mechanisms. Elevated Ca^2+^ levels, both at cytosolic and mitochondrial levels, become detrimental with increased noninhibited, non-scavenged, or nondegraded oxidants [46]. Hence, we decided to check antioxidant enzyme expressions at mRNA level to examine whether ROS scavenging produced during neuroinflammation was protective.

Many of the biochemical and molecular factors that are upregulated during neuronal cell death or apoptosis induction are not uniformly observed and vary with stimulants and the severity of cellular stress [47]. One such observation demonstrated that post-inflammatory responses mediated by NF-κB were the altered expression of oxidant-scavenging enzymes such as SOD1, CAT, and GPx. From our results, it was evident that, except for SOD1, we did not observe an increase in CAT or GPx activity as reported by many studies [48,49]. We may have missed the exact time points of expression of such oxidant scavengers, or a missing link that is transcriptionally controlling antioxidant-enzyme expression. However, SOD1 expression in our study was in agreement with other published reports in the literature [50,51] and clearly demonstrated the production of superoxide O_2_^−^ and the need for its catalytic degradation into H_2_O_2_ and O_2_. This further explains the possible relationship and involvement of mitochondrial dysfunction, thus promoting apoptosis in neuroinflammation.

### 4.3. MSCs Coculture Rescues Stressed Neuronal Cells from Apoptosis

Our initial study objective was to assess whether MSCs cocultures would rescue stressed cells to the extent that they withstood injury associated with reperfusion (reoxygenation) due to therapeutic measures. MSCs are well-known for their modulating activities [52] especially through either direct integration [53] or secreting molecular signals, which, in turn, transcriptionally regulate the host system to sustain stress signals [54]. In many instances, we do not know exactly how MSCs prime-stressed cells; however, MSCs, which were added at the end of oxidative-stress stimulant episodes, clearly and rapidly rescued stressed cells rather than the regular conditioning medium used for longer periods. Further cell loss was relatively lower when compared with higher cellular loss observed with a conditioning medium [55]. In our case, MSCs cocultures resulted in the rescue of anti-inflammatory cytokines, which were diminished during stress. This was well noted in many other studies and it is key to understanding that rescue is initiated through the upregulation of anti-inflammatory cytokines. On the other hand, downregulation of pro-inflammatory cytokines, transcriptional inducer NF-κB, and downstream COX-2 is evidence of cell restoration from the apoptotic phenotype [56]. It has to be noted that an in vitro coculture does not fully satisfy in vivo conditions and we could not thereby foresee complete reversal of neuronal cells to normal levels at the given experimental time points. It has to be further noted that MSCs transplantation or mobilization in real time signals the regeneration of the damaged cells or initiates the repair process [57]. Some coculture studies with other cell types, like activated macrophages, diminished the expression of IL-12 and TNF-α which was well in agreement with our observations.

Downregulation of NF-κB expression and, thereby, reversal of the inflammatory factors observed in our study can be linked to MSCs activation by proinflammatory cytokines and the stem-cell factor (SCF) [58,59]. It is of note that the SCF is neuroprotective and known to regulate expression of antiapoptotic proteins, such as Bcl-2 and Bcl-xl [58]. Furthermore, the link between NF-κB and COX-2 observed in our study was totally mediated by MSCs inhibiting COX-2-dependent cell death. The evidence for rescue from an inflammatory phenotype was observed due to the reduction in apoptotic-cell accumulation and, thereby, cell death during the MSCs coculture. In the inflammation pathway, the role of oxidant-degrading enzymes is an important milestone, which is well-regulated by mediators like cytokines and transcriptional regulators. Our results showed that only SOD1 is active and expressed at mRNA levels, while CAT and GPx did not show any significant changes, even in MSCs cocultures. It is well known from the literature that SOD1 is the most abundant and powerful antioxidant enzyme catalyzing ROS conversion along with completing the action of CAT [50]. Although all three enzymes were observed in all cells, we did not observe any significant changes in CAT and GPx. This may be due to antioxidant compensation in MSCs resulting in non-expression of the endogenous CAT and GPx enzymes, or simply that SOD1 supersedes the other antioxidants.

To substantiate the reversal claim, the MSCs coculture drastically reduced basal elevated [Ca^2+^]_i_ and, subsequently, ROS. Inflammatory-cell rescue to normal or near-normal levels has been reported previously by many studies, including ours [10,60,61,62] with more emphasis on mitochondrial involvement. It should be noted that ROS and calcium interactions are bidirectional, as calcium is essential for ROS promotion, while the latter are a potent regulator of calcium signals [63]. Addressing the functional nature of neuronal cells, and the entire inflammatory process being Ca^2^^+^-dependent, we showed serotonin release that was proportional to [Ca^2+^]_i_ homeostasis. It is well known that serotonin exocytosis, in addition to its neurotransmitter functions, is dependent on cellular Ca^2^^+^ levels. Physiologically low serotonin is associated with depression, while excessive expression of serotonin is taken up by surrounding glia in vivo [64,65]. Our results also showed serotonin release was a Ca^2^^+^-dependent process with [Ca^2+^]_i_ restoration. Controlling ROS with MSCs drastically reduced their release, indicating that the cells that were stressed upon, producing more neurotransmitters, had returned to physiological levels.

## 5. Conclusions

It was evident that MSCs cocultured with stressed neuronal cells can alleviate the harmful effect of ischemia-mediated oxidative stress and apoptosis. MSCs function by promoting anti-inflammatory cytokines and improving the expression of oxidant-scavenging enzymes, such as SOD1. Further inflammatory phenotype rescue was characterized by the reversal and restoration of inflammatory mediators such as NF-κB, COX–2, Ca^2+^, and ROS in MSCs coculture experiments. The control of elevated serotonin release against [Ca^2+^]_i_ homeostasis curbed by MSCs provides insight into the functional restoration of neuronal cells.

## Supplementary Materials

The following are available online at http://www.mdpi.com/2073-4409/7/12/250/s1, Figure S1: Pre and post enrichment of MSCs, Figure S2: Expression of Tuji1 and NeuN 12 days’ post differentiation.

## References

[1] Lin W., Hsuan Y.C., Lin M.T., Kuo T.W., Lin C.H., Su Y.C., Niu K.C., Chang C.P., Lin H.J. (2017). Human Umbilical Cord Mesenchymal Stem Cells Preserve Adult Newborn Neurons and Reduce Neurological Injury after Cerebral Ischemia by Reducing the Number of Hypertrophic Microglia/Macrophages. Cell Transplant..

[2] Ikonomidou C., Kaindl A.M. (2011). Neuronal Death and Oxidative Stress in the Developing Brain. Antioxid. Redox Signal..

[3] Martin L.J. (2010). Mitochondrial and Cell Death Mechanisms in Neurodegenerative Diseases. Pharmaceuticals.

[4] Lakhan S.E., Kirchgessner A., Hofer M. (2009). Inflammatory mechanisms in ischemic stroke: therapeutic approaches. J. Transl. Med..

[5] Gorman A.M. (2008). Neuronal cell death in neurodegenerative diseases: recurring themes around protein handling. J. Cell. Mol. Med..

[6] Baraniak P.R., McDevitt T.C. (2010). Stem cell paracrine actions and tissue regeneration. Regen. Med..

[7] Bergstrom T., Forsberg-Nilsson K. (2012). Neural stem cells: Brain building blocks and beyond. Ups. J. Med. Sci..

[8] Jellinger K.A. (2009). Recent advances in our understanding of neurodegeneration. J. Neural Transm..

[9] Kalogeris T., Baines C.P., Krenz M., Korthuis R.J. (2012). Cell biology of ischemia/reperfusion injury. Int. Rev. Cell Mol. Biol..

[10] Gorlach A., Bertram K., Hudecova S., Krizanova O. (2015). Calcium and ROS: A mutual interplay. Redox Biol..

[11] Doonan P.J., Chandramoorthy H.C., Hoffman N.E., Zhang X., Cardenas C., Shanmughapriya S., Rajan S., Vallem S., Chen X., Foskett J.K. (2014). LETM1-dependent mitochondrial Ca2+ flux modulates cellular bioenergetics and proliferation. FASEB J..

[12] Foskett J.K., Madesh M. (2014). Regulation of the mitochondrial Ca(2+) uniporter by MICU1 and MICU2. Biochem. Biophys. Res. Commun..

[13] Wang W.Y., Tan M.S., Yu J.T., Tan L. (2015). Role of pro-inflammatory cytokines released from microglia in Alzheimer’s disease. Ann. Transl. Med..

[14] Chitnis T., Weiner H.L. (2017). CNS inflammation and neurodegeneration. J. Clin. Invest..

[15] Dahm T., Rudolph H., Schwerk C., Schroten H., Tenenbaum T. (2016). Neuroinvasion and Inflammation in Viral Central Nervous System Infections. Mediators Inflamm..

[16] Zundorf G., Reiser G. (2011). Calcium dysregulation and homeostasis of neural calcium in the molecular mechanisms of neurodegenerative diseases provide multiple targets for neuroprotection. Antioxid. Redox Signal..

[17] Scheibe F., Klein O., Klose J., Priller J. (2012). Mesenchymal stromal cells rescue cortical neurons from apoptotic cell death in an in vitro model of cerebral ischemia. Cell. Mol. Neurobiol..

[18] Mahrouf-Yorgov M., Augeul L., Da Silva C.C., Jourdan M., Rigolet M., Manin S., Ferrera R., Ovize M., Henry A., Guguin A. (2017). Mesenchymal stem cells sense mitochondria released from damaged cells as danger signals to activate their rescue properties. Cell Death Differ..

[19] Shipley M.M., Mangold C.A., Szpara M.L. (2016). Differentiation of the SH-SY5Y Human Neuroblastoma Cell Line. J. Vis. Ex..

[20] Messi E., Florian M.C., Caccia C., Zanisi M., Maggi R. (2008). Retinoic acid reduces human neuroblastoma cell migration and invasiveness: Effects on DCX, LIS1, neurofilaments-68 and vimentin expression. BMC Cancer.

[21] Chandramoorthy H.C., Bin-Jaliah I., Karari H., Rajagopalan P., Ahmed Shariff M.E., Al-Hakami A., Al-Humayad S.M., Baptain F.A., Ahmed H.S., Yassin H.Z. (2018). MSCs ameliorates DPN induced cellular pathology via [Ca(2+)]i homeostasis and scavenging the pro-inflammatory cytokines. J. Cell. Physiol..

[22] Chen J., Guo Y., Cheng W., Chen R., Liu T., Chen Z., Tan S. (2013). High glucose induces apoptosis and suppresses proliferation of adult rat neural stem cells following in vitro ischemia. BMC Neurosci..

[23] Borradaile N.M., Han X., Harp J.D., Gale S.E., Ory D.S., Schaffer J.E. (2006). Disruption of endoplasmic reticulum structure and integrity in lipotoxic cell death. J. Lipid Res..

[24] Irrinki K.M., Mallilankaraman K., Thapa R.J., Chandramoorthy H.C., Smith F.J., Jog N.R., Gandhirajan R.K., Kelsen S.G., Houser S.R., May M.J. (2011). Requirement of FADD, NEMO, and BAX/BAK for aberrant mitochondrial function in tumor necrosis factor alpha-induced necrosis. Mol. Cell. Biol..

[25] Mallilankaraman K., Cardenas C., Doonan P.J., Chandramoorthy H.C., Irrinki K.M., Golenar T., Csordás G., Madireddi P., Yang J., Müller M. (2012). MCUR1 is an essential component of mitochondrial Ca2+ uptake that regulates cellular metabolism. Nat. Cell Biol..

[26] Jahn K., Wieltsch C., Blumer N., Mehlich M., Pathak H., Khan A.Q., Hildebrandt H., Frieling H. (2017). A cell culture model for investigation of synapse influenceability: Epigenetics, expression and function of gene targets important for synapse formation and preservation in SH-SY5Y neuroblastoma cells differentiated by retinoic acid. J. Neural Transm..

[27] Lopes F.M., da Motta L.L., De Bastiani M.A., Pfaffenseller B., Aguiar B.W., de Souza L.F., Zanatta G., Vargas D.M., Schönhofen P., Londero G.F. (2017). RA Differentiation Enhances Dopaminergic Features, Changes Redox Parameters, and Increases Dopamine Transporter Dependency in 6-Hydroxydopamine-Induced Neurotoxicity in SH-SY5Y Cells. Neurotox. Res..

[28] Cheung Y.T., Lau W.K., Yu M.S., Lai C.S., Yeung S.C., So K.F., Chang R.C.C. (2009). Effects of all-trans-retinoic acid on human SH-SY5Y neuroblastoma as in vitro model in neurotoxicity research. Neurotoxicology.

[29] Korecka J.A., van Kesteren R.E., Blaas E., Spitzer S.O., Kamstra J.H., Smit A.B., Swaab D.F., Verhaagen J., Bossers K. (2013). Phenotypic characterization of retinoic acid differentiated SH-SY5Y cells by transcriptional profiling. PLoS ONE.

[30] Neirinckx V., Coste C., Rogister B., Wislet-Gendebien S. (2013). Concise review: Adult mesenchymal stem cells, adult neural crest stem cells, and therapy of neurological pathologies: A state of play. Stem Cells Transl. Med..

[31] Kaltschmidt B., Widera D., Kaltschmidt C. (2005). Signaling via NF-κB in the nervous system. BBA Mol. Cell Res..

[32] Ben-Neriah Y., Karin M. (2011). Inflammation meets cancer, with NF-κB as the matchmaker. Nat. Immun..

[33] Chen L.-F., Greene W.C. (2004). Shaping the nuclear action of NF-κB. Nat. Rev. Mol. Cell Biol..

[34] Koo J.W., Russo S.J., Ferguson D., Nestler E.J., Duman R.S. (2010). Nuclear factor-κB is a critical mediator of stress-impaired neurogenesis and depressive behavior. Proc. Natl. Acad. Sci. USA.

[35] Lanzillotta A., Sarnico I., Ingrassia R., Boroni F., Branca C., Benarese M., Faraco G., Blasi F., Chiarugi A., Spano P. (2010). The acetylation of RelA in Lys310 dictates the NF-κB-dependent response in post-ischemic injury. Cell Death Dis..

[36] Smith W.L., Garavito R.M., DeWitt D.L. (1996). Prostaglandin endoperoxide H synthases (cyclooxygenases)-1 and -2. J. Biol. Chem..

[37] Planas A.M., Soriano M.A., Rodriguez-Farre E., Ferrer I. (1995). Induction of cyclooxygenase-2 mRNA and protein following transient focal ischemia in the rat brain. Neurosci. Lett..

[38] Krause D.L., Muller N. (2010). Neuroinflammation, microglia and implications for anti-inflammatory treatment in Alzheimer’s disease. Int. J. Alzheimers Dis..

[39] Hirst W.D., Young K.A., Newton R., Allport V.C., Marriott D.R., Wilkin G.P. (1999). Expression of COX-2 by normal and reactive astrocytes in the adult rat central nervous system. Mol. Cell. Neurosci..

[40] Tan Q., Wang M., Yu M., Zhang J., Bristow R.G., Hill R.P., Tannock I.F. (2016). Role of Autophagy as a Survival Mechanism for Hypoxic Cells in Tumors. Neoplasia.

[41] Facchinetti F., Dawson V.L., Dawson T.M. (1998). Free radicals as mediators of neuronal injury. Cell. Mol. Neurobiol..

[42] Pinton P., Giorgi C., Siviero R., Zecchini E., Rizzuto R. (2008). Calcium and apoptosis: ER-mitochondria Ca2+ transfer in the control of apoptosis. Oncogene.

[43] Haidara M.A., Assiri A.S., Youssef M.A., Mahmoud M.M., Ahmed M.S.E., Al-Hakami A., Chandramoorthy H.C. (2015). Differentiated mesenchymal stem cells ameliorate cardiovascular complications in diabetic rats. Cell Tissue Res..

[44] Mallilankaraman K., Doonan P., Cardenas C., Chandramoorthy H.C., Muller M., Miller R., Hoffman N.E., Gandhirajan R.K., Molgó J., Birnbaum M.J. (2012). MICU1 is an essential gatekeeper for MCU-mediated mitochondrial Ca(2+) uptake that regulates cell survival. Cell.

[45] Clapham D.E. (2007). Calcium signaling. Cell.

[46] Celsi F., Pizzo P., Brini M., Leo S., Fotino C., Pinton P., Rizzuto R. (2009). Mitochondria, calcium and cell death: A deadly triad in neurodegeneration. Biochim. Biophys. Acta.

[47] Jellinger K.A. (2010). Basic mechanisms of neurodegeneration: A critical update. J. Cell. Mol. Med..

[48] Warner D.S., Sheng H., Batinic-Haberle I. (2004). Oxidants, antioxidants and the ischemic brain. J. Exp. Biol..

[49] Shirley R., Ord E.N., Work L.M. (2014). Oxidative Stress and the Use of Antioxidants in Stroke. Antioxid..

[50] Fukai T., Ushio-Fukai M. (2011). Superoxide dismutases: Role in redox signaling, vascular function, and diseases. Antioxid. Redox Signal..

[51] Fattman C.L., Schaefer L.M., Oury T.D. (2003). Extracellular superoxide dismutase in biology and medicine. Free Radic. Biol. Med..

[52] Yagi H., Soto-Gutierrez A., Parekkadan B., Kitagawa Y., Tompkins R.G., Kobayashi N., Yarmush M.L. (2010). Mesenchymal stem cells: Mechanisms of immunomodulation and homing. Cell Transplant..

[53] Spees J.L., Lee R.H., Gregory C.A. (2016). Mechanisms of mesenchymal stem/stromal cell function. Stem Cell Res. Ther..

[54] Sobacchi C., Palagano E., Villa A., Menale C. (2017). Soluble Factors on Stage to Direct Mesenchymal Stem Cells Fate. Front. Bioeng. Biotechnol..

[55] Kadekar D., Rangole S., Kale V., Limaye L. (2016). Conditioned Medium from Placental Mesenchymal Stem Cells Reduces Oxidative Stress during the Cryopreservation of Ex Vivo Expanded Umbilical Cord Blood Cells. PLoS ONE.

[56] Kim J., Vaish V., Feng M., Field K., Chatzistamou I., Shim M. (2016). Transgenic expression of cyclooxygenase-2 (COX2) causes premature aging phenotypes in mice. Aging.

[57] Stonesifer C., Corey S., Ghanekar S., Diamandis Z., Acosta S.A., Borlongan C.V. (2017). Stem cell therapy for abrogating stroke-induced neuroinflammation and relevant secondary cell death mechanisms. Prog. Neurobiol..

[58] Dhandapani K.M., Wade F.M., Wakade C., Mahesh V.B., Brann D.W. (2005). Neuroprotection by stem cell factor in rat cortical neurons involves AKT and NFkappaB. J. Neurochem..

[59] Saldana L., Valles G., Bensiamar F., Mancebo F.J., Garcia-Rey E., Vilaboa N. (2017). Paracrine interactions between mesenchymal stem cells and macrophages are regulated by 1,25-dihydroxyvitamin D3. Sci. Rep..

[60] Chaudhari N., Talwar P., Parimisetty A., Lefebvre d’Hellencourt C., Ravanan P. (2014). A molecular web: Endoplasmic reticulum stress, inflammation, and oxidative stress. Front. Cell. Neurosci..

[61] Redza-Dutordoir M., Averill-Bates D.A. (2016). Activation of apoptosis signalling pathways by reactive oxygen species. Biochim. Biophys. Acta..

[62] Ambudkar I.S., Muallem S. (2016). ROS and Ca(2^+^)-Partners in sickness and in health. Cell Calcium.

[63] Gordeeva A.V., Zvyagilskaya R.A., Labas Y.A. (2003). Cross-talk between reactive oxygen species and calcium in living cells. Biochemistry.

[64] Leon-Pinzon C., Cercos M.G., Noguez P., Trueta C., De-Miguel F.F. (2014). Exocytosis of serotonin from the neuronal soma is sustained by a serotonin and calcium-dependent feedback loop. Front. Cell. Neurosci..

[65] De-Miguel F.F., Leon-Pinzon C., Noguez P., Mendez B. (2015). Serotonin release from the neuronal cell body and its long-lasting effects on the nervous system. Philos. Trans. R. Soc. Lond. B Biol. Sci..

