# Supplementary material for: Mesenchymal Stem Cells (MSCs) Coculture Protects [Ca2+]i Orchestrated Oxidant Mediated Damage in Differentiated Neurons In Vitro
Source: Cells. 2018 Dec 6;7(12):250. doi: 10.3390/cells7120250 (PMC6315478; doi:10.3390/cells7120250)
Supplement: Supplementary file 1 [file cells-07-00250-s001.zip › Supplimentary Figures 06122018/Supplementary Material.docx]

**Supplementary Material**

**
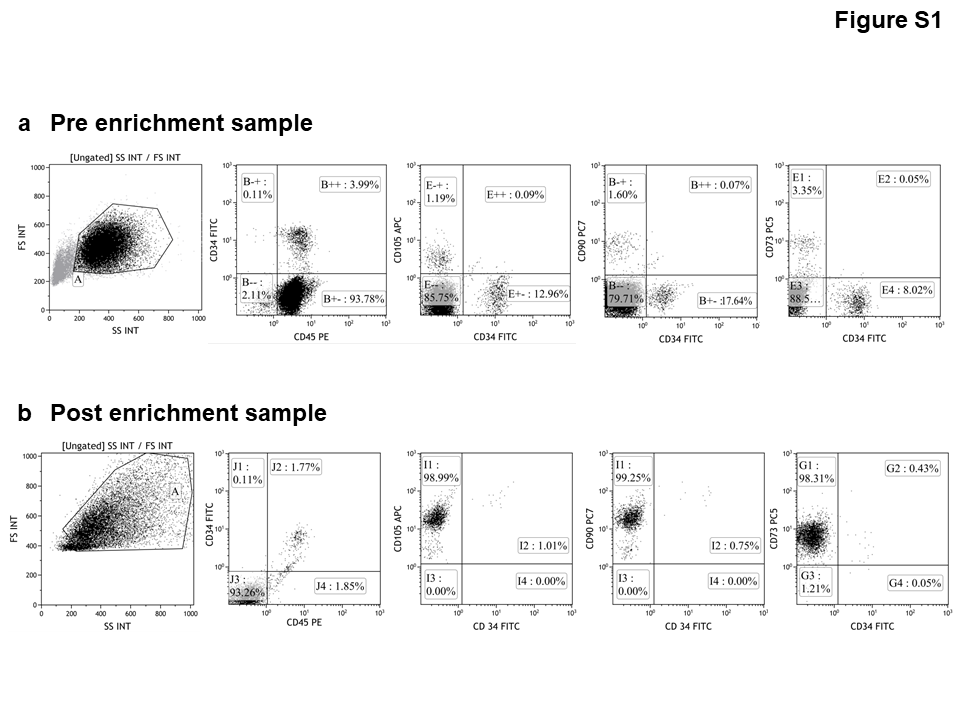
**

**Figure S1.** Pre- and post-enrichment of MSCs. (**a**) Pre-enrichment UCB samples showing cells CD45+, CD34+, CD105+, CD90+ CD73+ cells. (**b**) Post enrichment for CD105+CD90+CD73 positive selection is devoid of CD45+ and CD34+ cells.

**
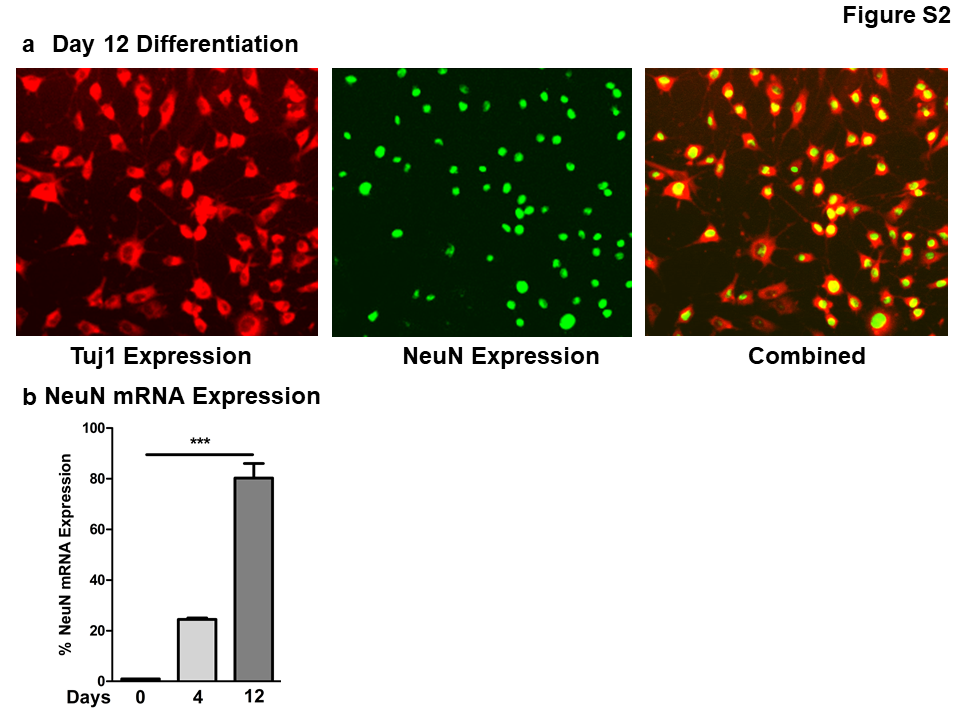
**

**Figure S2.** Expression of Tuji1 and NeuN 12 days’ post differentiation. (**a**) Day 12 differentiation and staining of Tuj1 and NeuN and combined. (**b**) NeuN mRNA expression of 4^th^ and 12^th^ day by RT-PCR.
