# Supplementary figures and images for: Mesenchymal Stem Cells (MSCs) Coculture Protects [Ca2+]i Orchestrated Oxidant Mediated Damage in Differentiated Neurons In Vitro
Source: Cells. 2018 Dec 6;7(12):250. doi: 10.3390/cells7120250 (PMC6315478; doi:10.3390/cells7120250)

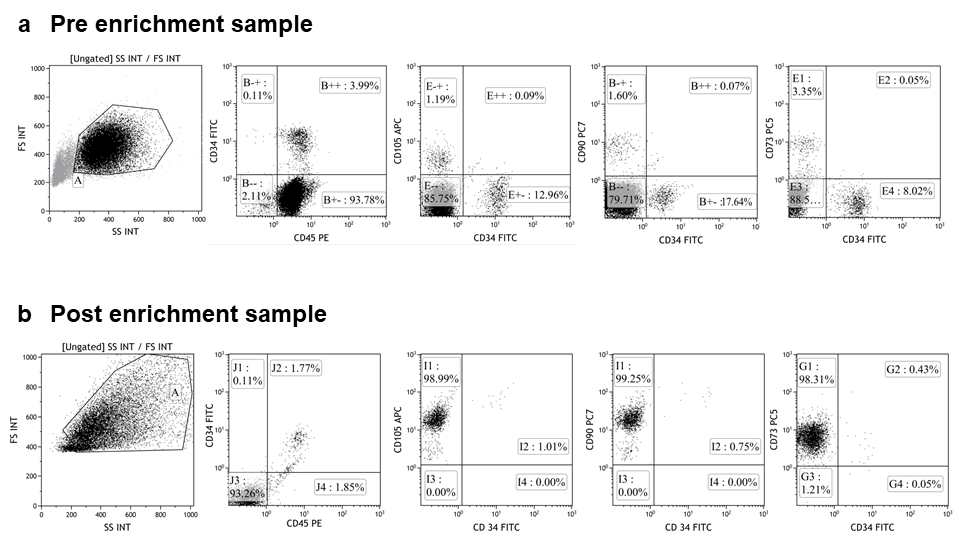

Supplement: Supplementary file 1 [file cells-07-00250-s001.zip › Supplimentary Figures 06122018/SF1.TIF]

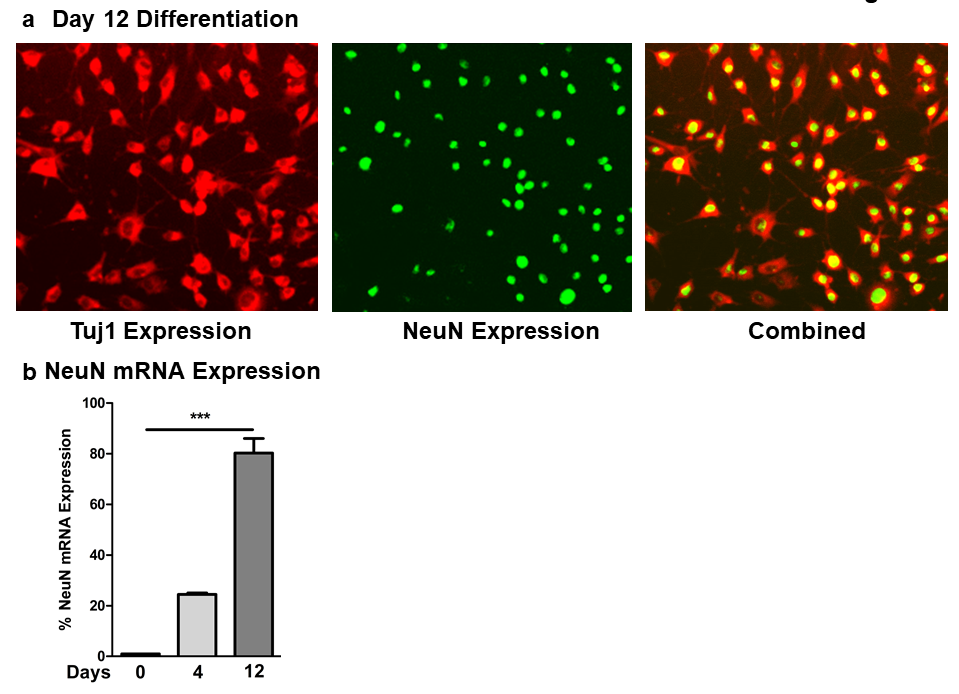

Supplement: Supplementary file 1 [file cells-07-00250-s001.zip › Supplimentary Figures 06122018/SF2.TIF]
